# Elevated Neutrophil Gelatinase-Associated Lipocalin Is Associated With the Severity of Kidney Injury and Poor Prognosis of Patients With COVID-19

**DOI:** 10.1016/j.ekir.2021.09.005

**Published:** 2021-10-08

**Authors:** Katherine Xu, Ning Shang, Abraham Levitman, Alexa Corker, Satoru Kudose, Andrew Yaeh, Uddhav Neupane, Jacob Stevens, Rosemary Sampogna, Angela M. Mills, Vivette D’Agati, Sumit Mohan, Krzysztof Kiryluk, Jonathan Barasch

**Affiliations:** 1 Department of Medicine, Columbia University, New York, New York, USA; 2 Department of Pathology, Columbia University, New York, New York, USA; 3 Department of Emergency Medicine, Columbia University, New York, New York, USA; 4 Department of Epidemiology, Columbia University, New York, New York, USA

**Keywords:** acute kidney injury, acute tubular injury, COVID-19, dialysis, kidney biopsy, KIM1, NGAL

## Abstract

**Introduction:**

Loss of kidney function is a common feature of COVID-19 infection, but serum creatinine (SCr) is not a sensitive or specific marker of kidney injury. We tested whether molecular biomarkers of tubular injury measured at hospital admission were associated with acute kidney injury (AKI) in those with COVID-19 infection.

**Methods:**

This is a prospective cohort observational study consisting of 444 consecutive patients with SARS-CoV-2 enrolled in the Columbia University emergency department (ED) at the peak of the pandemic in New York (March 2020–April 2020). Urine and blood were collected simultaneously at hospital admission (median time: day 0, interquartile range: 0–2 days), and urine biomarkers were analyzed by enzyme-linked immunosorbent assay (ELISA) and a novel dipstick. Kidney biopsies were probed for biomarker RNA and for histopathologic acute tubular injury (ATI) scores.

**Results:**

Admission urinary neutrophil gelatinase-associated lipocalin (uNGAL) level was associated with AKI diagnosis (267 ± 301 vs. 96 ± 139 ng/ml, *P* < 0.0001) and staging; uNGAL levels >150 ng/ml had 80% specificity and 75% sensitivity to diagnose AKI stages 2 to 3. Admission uNGAL level quantitatively associated with prolonged AKI, dialysis, shock, prolonged hospitalization, and in-hospital death, even when admission SCr level was not elevated. The risk of dialysis increased almost 4-fold per SD of uNGAL independently of baseline SCr, comorbidities, and proteinuria (odds ratio [OR] [95% CI]: 3.59 [1.83–7.45], *P* < 0.001). In the kidneys of those with COVID-19, NGAL mRNA expression broadened in parallel with severe histopathologic injury (ATI). Conversely, low uNGAL levels at admission ruled out stages 2 to 3 AKI (negative predictive value: 0.95, 95% CI: 0.92–0.97) and the need for dialysis (negative predictive value: 0.98, 95% CI: 0.96–0.99). Although proteinuria and urinary (u)KIM-1 were implicated in tubular injury, neither was diagnostic of AKI stages.

**Conclusion:**

In the patients with COVID-19, uNGAL level was quantitatively associated with histopathologic injury (ATI), loss of kidney function (AKI), and severity of patient outcomes.

Acute loss of kidney function, measured by a rise in SCr, is common in the setting of acute SARS-CoV-2 infection.^1^, ^2^, ^3^, ^4^ Elevated SCr level is present in one-third of the patients hospitalized with COVID-19.^10^, ^11^, ^5^, ^6^, ^7^, ^8^, ^9^ Yet, the measurement of SCr fails to reflect the full burden of COVID-19 kidney injury. For example, SCr can only detect kidney dysfunction in retrospect, after enough time has elapsed for the accumulation of SCr to a diagnostic threshold. In addition, clinically significant changes in SCr may not occur in subtotal or focal kidney damage as a result of compensatory changes in uninjured nephrons which can attenuate the rise in SCr level. Even when elevations in SCr level are apparent, evidence of tubular injury may be lacking, for example in the presence of volume depletion,^12^^,^^13^ a common presentation in patients with COVID-19–associated diarrhea.^10^^,^^14^ A rise in SCr level may also be confounded by the incidence of rhabdomyolysis in COVID-19, which enhances creatinine production.^15^^,^^16^

The burden of kidney injury during surges of COVID-19 has strained hospital resources, including Emergency Medicine, Critical Care, and Nephrology, creating an urgent need to limit delays in the identification of individuals at risk for kidney injury, loss of kidney function, and renal replacement therapy. As a result, triage decisions for patients and for resource allocations would benefit from the use of a rapidly responsive, sensitive, and specific noninvasive marker of kidney injury and its attendant outcomes common in COVID-19 infection.

Previous research in human and mouse models revealed that 2 molecular markers of tubular injury, uNGAL and uKIM-1, were derived from different segments of the kidney and allow for sensitive detection and real-time distinction between volume sensitive and volume insensitive intrinsic forms of tubular injury.^12^^,^^17^, ^18^, ^19^ Previous evidence of the utility of these markers in the detection of AKI on presentation to the ED^12^^,^^20^, ^21^, ^22^ raises the question of whether they might be of value in patients with COVID-19. The need for new diagnostic testing is particularly evident because many patients with COVID-19 are presenting to the hospital for the first time and do not have previous records of SCr.

Here, we study the performance of uNGAL and uKIM-1 in a large cohort of patients with acute COVID-19 presenting to the Columbia University ED at the peak of the pandemic in New York City (March 2020–April 2020). We tested the association of uNGAL and uKIM-1 with the subsequent diagnosis, duration, and severity of AKI as defined by the Acute Kidney Injury Network (AKIN) criteria and with in-hospital death, dialysis, shock, respiratory failure, and length of hospital stay. We used both standard ELISA methods and a novel dipstick that can measure uNGAL at the bedside. Finally, we probed whether NGAL and KIM-1 RNA correlated with histopathologic metrics of ATI in kidney biopsy findings from the patients with COVID-19.

## Methods

### Human Subjects

**Figure 1:**
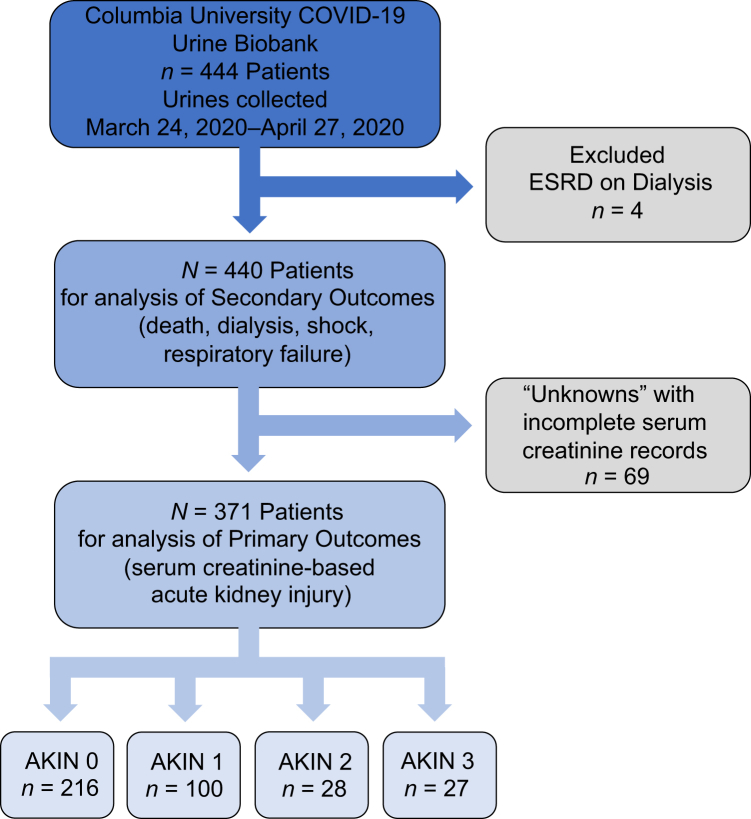
Urine sample collection: Residual urine samples were collected from 444 consecutive patients with COVID-19 by the Columbia University COVID-19 Urine Biobank between March 24, 2020 and April 27, 2020. A total of 4 patients with end-stage kidney disease on dialysis were excluded from the final cohort (*N* = 440). Patients with complete records of SCr measurements were staged by AKIN criteria *(n* = 371). A total of 69 patients missing adequate kidney function data were labeled “unknown.” AKIN, Acute Kidney Injury Network**;** SCr, serum creatinine.

The Columbia COVID-19 Biobank recruited consecutive COVID-19 cases (positive result from nasopharyngeal SARS-CoV-2 polymerase chain reaction test), regardless of age, sex, or race/ethnicity who received care at the Columbia University Irving Medical Center. The Biobank stored residual blood and urine samples after clinical testing from every patient with COVID-19 including an initial urine sample and serum obtained at hospital admission, which were used for biomarker measurements. For our analysis, we identified 444 consecutive patients who were admitted between March 24, 2020, and April, 27, 2020, with COVID-19. This included 371 patients who had not only a urine sample from presentation but also information on baseline SCr and complete SCr measurements in the hospital required to determine the stage and duration of AKI (Figure 1). In addition, 4 patients with end-stage renal disease were excluded.

Kidney biopsies were accessioned by the Columbia University Irving Medical Center Renal Pathology Laboratory, including 13 kidney biopsies from COVID-19 cases^23^ and 4 non–COVID-19 specimens analyzed by *in situ* hybridization for NGAL and KIM-1 RNA.

The COVID (+) cohort was compared with a COVID (−) cohort that was recruited in an identical fashion in the Columbia University Irving Medical Center ED between June 2017 and January 2019.^20^ Similar to the COVID (+) cohort, 426 consecutive COVID (−) patients were enrolled at presentation, regardless of age, sex, or race/ethnicity. This included 318 patients with complete SCr measurements in the hospital required to determine stage and duration of AKI. Urine samples were collected within 24 hours of arrival in the ED. Patients with end-stage renal disease were excluded.

### Definition of Primary Outcomes

Primary outcomes included SCr-based AKI, AKIN stage, and the duration of elevated SCr level consistently in both the COVID (+) and the COVID (−) cohorts. In both cases, baseline SCr level was defined using a standardized algorithm, as previously described by Stevens *et al.*,^20^ in the following order:

• Median SCr from 365 to 31 days before urine collection. If not available, then:
• Minimum SCr from 30 days before urine collection to the day of collection. If not available, then:
• Minimum SCr from urine collection to 7 days in the hospital.

Patients were classified as “Unknowns” if none of the above-mentioned criteria were met.

AKIN^23^ stages were classified as follows:

• AKIN stage 1: ≥0.3 mg/dl increase in SCr within a 48-hour window OR 1.5- to 2-fold increase in SCr compared with baseline.
• AKIN stage 2: >2- to 3-fold increase in SCr compared with baseline.
• AKIN stage 3: ≥0.5 mg/dl increase in SCr within a 48-hour window when SCr ≥4.0 mg/dl OR >3-fold increase in SCr compared with baseline.

The peak SCr level measured within 48 hours after urine collection was used to diagnose AKIN stage. In select cases, the day 1 AKIN score was imputed when the preceding and subsequent AKIN scores were identical.

Further categorization was based on the duration of SCr elevation above the baseline:

• No AKI (AKIN 0)—not meeting AKIN criteria within 2 days of presentation (must have SCr values for both days).
• Transient AKI—met AKIN criteria on day 0 or 1 of presentation but normalized below AKIN detection thresholds within 2 days after first detection (total AKI duration <72 hours).
• Sustained AKI (sAKI)—met AKIN criteria within 2 days of presentation but normalized below the AKIN detection thresholds only after 2 days from the first detection (total AKI duration >72 hours).
• Unknown—missing baseline SCr, or insufficient SCr measurements to determine SCr kinetics, or missing measurements on day 0 or 1 that could not be imputed owing to discrepant AKIN scores (Figure 1). Further categorization of elevated SCr level was based on the duration of SCr elevation above the baseline, including transient AKI rapidly normalizing SCr level (<72 hours) and sustained (sAKI) delayed normalization of SCr level (>72 hours).

Urine output was not used for AKI definition because of the variable use of Foley catheters and incomplete recording of urine output in the ED.

### Definition of Secondary Outcomes

Secondary outcomes included dialysis, shock, respiratory failure, length of hospital stay, and in-hospital death. Shock was defined by the need for vasopressors, and respiratory failure was defined by the need for either invasive or noninvasive positive pressure ventilation. Staging of chronic kidney disease was determined using the Chronic Kidney Disease Epidemiology Collaboration estimated glomerular filtration rate formula and the baseline creatinine as described previously.^24^

### Laboratory Analysis

All urinary measurements were blinded to clinical data. NGAL and uKIM-1 were measured by ELISA (KIM-1: Enzo, ADI-900-226-0001; NGAL: BioPorto, KIT036). Proteinuria was detected with Chemstrip 10 SG (Roche Diagnostics). Urine was centrifuged (12,000 rpm; 10 minutes) and applied to NGAL gRAD dipsticks (BioPorto), and color development was compared with a color scale marking NGAL (ng/ml) concentration by 2 independent readers. Urinary cell pellets were analyzed for LRP2 and UMOD by immunoblot with SDS-PAGE (Bio-Rad Laboratories), rabbit anti-LRP2 (1:1000, Abcam, ab76969), sheep anti-UMOD (1:2000, Meridian Life Science, K90071C), and polyclonal secondary antibodies conjugated to HRP (1:10,000, Jackson ImmunoResearch).

### Histologic Identification of ATI

Histologic features of ATI included loss of brush border, epithelial simplification, intracytoplasmic vacuolization, overt necrosis, apoptosis, and cell shedding. A semiquantitative scale was used to measure the extent of ATI, which was as follows: none (<5% of tubules involved), mild (<25%), moderate (25%–50%), and severe (>50%).

### Pathologic Analysis

*In situ* hybridization on formalin-fixed, paraffin-embedded human kidneys used the chromogenic RNAscope 2.5 HD Duplex Reagent Kit (Advanced Cell Diagnostics, 322430). Probes included NGAL: Hs-LCN2 (559441) and Hs-LCN2-C2 (559441-C2 at 1:600); KIM-1: Hs-HAVCR1-O1 (538081 and 538081-C2 at 1:100); and aquaporin 2: Hs-AQP2 (434861) and Hs-LRP2 (532391).

### Confirmatory Renal Ischemia–Reperfusion Injury Analysis

Male and female wild-type C57Bl/6 mice, aged 8 to 10 weeks (Jackson Labs) were anesthetized with isoflurane and placed on a warming table (rectal temperature: 37 °C). Microvascular clamps (Fine Science Tools) were applied to the left renal pedicle for 10, 20, 30, or 40 minutes. The kidneys were harvested at 24 hours. *In situ* hybridization on formalin-fixed, paraffin-embedded kidneys used the NGAL probe: Mm-Lcn2-C2 (313971-C2) and the KIM-1 probe: Mm-Havcr1 (472551).

### Statistical Analysis

Normality of continuous variables was tested using Shapiro–Wilk test. Normally distributed continuous variables were compared using a 2-sample *t* test and summarized as mean ± SD. Non-normally distributed continuous variables were summarized as medians and ranges and compared using nonparametric Mann–Whitney *U* test. To improve interpretability of effect estimates from multivariate regression models, all non-normally distributed predictors (including uNGAL and uKIM-1 levels) were log-transformed and standard-normalized before statistical testing. The effect sizes for biomarkers were expressed per SD unit of normalized predictors. Categorical variables were compared using χ^2^ or Fisher exact test. For testing binary outcomes, we used logistic regression. Ordinal outcomes, such as AKIN stage, were tested using ordinal logistic regression. Ordinal predictors, such as urine dipstick category or proteinuria grade, were tested under the assumption of linear effects using a slope test within the framework of a generalized linear model tailored to the outcome of interest (e.g., logistic or ordinal logistic for binary or ordinal outcomes, respectively). We used Cox proportional hazards model for the time-to-event analyses for death outcome. We used competing risks regression model for the analysis of the hospital length of stay, with death as a competing risk.^25^^,^^26^ The proportional hazards assumption was verified by testing scaled Schoenfeld residuals for each predictor against observation time. Associations of urinary biomarkers with clinical outcomes were adjusted for the following covariates: age, sex, race, ethnicity (minimally adjusted model), baseline SCr, and preexisting obesity, diabetes, hypertension, transplant (any organ), cancers, cardiovascular disease (coronary artery disease, heart failure, cerebral infarction), pulmonary disease (asthma, chronic obstructive pulmonary disease, interstitial pulmonary disease, primary pulmonary hypertension, idiopathic pulmonary fibrosis) (fully adjusted model 1), and proteinuria (fully adjusted model 2). In the analysis of primary outcomes, we considered 2-sided *P* < 0.05 as statistically significant. In the analysis of secondary outcomes, we considered *P* < 0.01 as statistically significant (Bonferroni-corrected for 5 independent outcomes tested). We additionally stratified patients by both uNGAL (≥150 ng/ml) and SCr-based AKI into the following 4 groups: NGAL−AKI−, NGAL+AKI−, NGAL−AKI+, and NGAL+AKI+. We used χ^2^ test for pairwise comparisons of proportions with a clinical outcome between the groups and the Cochran-Armitage test for trend in proportions between low-risk (NGAL−AKI−), intermediate-risk (NGAL+AKI− or NGAL−AKI+), and high-risk (NGAL+AKI+) groups. Furthermore, each group was tested as a categorical predictor of outcome using logistic regression with low-risk (NGAL−AKI−) group as the reference. All statistical analyses were performed using R (CRAN version 4.0.4), including R add-on packages MASS (version 7.3-53.1), Odds Ratio (version 2.0.1), survival (version 3.2-7), cmprsk (version 2.2-10), and pROC (version 1.17. 0.1). Our study was reported according to Strengthening the Reporting of Observational studies in Epidemiology guidelines for cohort studies.^27^

### Oversight of Studies

The Columbia University Biobank COVID-19 studies were approved by the Columbia University Medical Center Institutional Review Board (AAAS7370 and AAAS7948). A subset of patients was included under a public health crisis institutional review board waiver of consent specifically for COVID-19 studies if patients were deceased, not able to consent, or for loss of contact.

Mice were used according to the Institutional Animal Care and Use Committee (AC-AAAY7464) and adhere to the National Institutes of Health Guide for the Care and Use of Laboratory Animals.

## Results

### Subjects

We analyzed urine samples from 444 ED patients with COVID-19 admitted for inpatient care. The samples were collected prospectively at a median time of day 0 (hospital admission, interquartile range: 0–2 days), within 1 day of a positive result from SARS-CoV-2 test in 70% of the patients (Figure 1). The cohort was diverse in age, sex, race, ethnicity (43.9% female, 20.5% African American, 54.1% Latinx), and preexisting comorbidities (Table 1). There were 4 patients with end-stage renal disease who were excluded from the study, and 69 patients had incomplete SCr records and were called “unknown” and analyzed separately (Figure 1).

**Table 1:** Baseline characteristics and outcomes for COVID-19–positive and –negative cohorts

Baseline Characteristics	COVID-19 (+) Cohort	COVID-19 (−) Cohort	*P* value
	*N* = 440	*N* = 426	
Age, mean (SD)	63.7 (19.2)	60.3 (17.9)	<0.05
Sex (%)			
Male	247 (56.1)	244 (57.3)	0.8, NS
Female	193 (43.9)	182 (42.7)	
Race (%)			
White or Caucasian	126 (28.6)	145 (34.0)	0.3, NS
Black or African American	90 (20.5)	77 (18.0)	
Asian	16 (3.6)	11 (3.0)	
Unknown	208 (47.2)	193 (45.0)	
Ethnicity (%)			
Hispanic or Latinx	238 (54.1)	104 (24.0)	<0.05
Non-Hispanic or non-Latinx	115 (26.1)	94 (22.0)	
Unknown	87 (19.8)	228 (54.0)	
Presenting comorbidities			
Obesity (%)	188 (42.7)	NA	
Hypertension (%)	143 (32.5)	NA	
Diabetes (%)	88 (20.0)	NA	
Cardiovascular disease (%)	49 (11.1)	NA	
Pulmonary disease (%)	52 (11.8)	NA	
Cancer (%)	62 (14.1)	NA	
Transplant (%)	28 (6.4)	NA	
Chronic kidney disease (%)			
No CKD or CKD 1–2	234 (53.2)	281 (66.0)	<0.05
CKD 3–5	170 (38.6)	145 (34.0)	
Kidney transplant	23 (5.2)	0 (0.0)	
Unknown	13 (3.0)	0 (0.0)	
Baseline SCr (mean (SD))	1.24 (1.22)	1.20 (0.91)	0.5, NS
Presenting proteinuria (%)			
Negative	57 (13.0)	142 (33.4)	<0.05
Trace	107 (24.5)	156 (36.7)	
1+	107 (24.5)	41 (9.6)	
2+	94 (21.5)	40 (9.4)	
3+	72 (16.5)	23 (5.4)	
Unknown	0 (0.0)	23 (5.4)	
Outcomes			
AKIN stage (%)			
AKIN 0	216 (49.1)	260 (61.0)	<0.05
AKIN 1	100 (22.7)	29 (6.8)	
AKIN 2	28 (6.4)	15 (3.5)	
AKIN 3	27 (6.1)	14 (3.3)	
Unknown	69 (15.7)	108 (25.4)	
AKI status (%)			
No AKI (AKIN 0)	216 (49.1)	260 (61)	<0.05
Transient AKI	67 (15.2)	39 (9.1)	
Sustained AKI	77 (17.5)	19 (4.5)	
Unknown	80 (18.2)	108 (25.4)	
Death within 90 d (%)	117 (26.6)	42 (9.9)	<0.05
Dialysis (%)	24 (5.5)	9 (2.1)	<0.05
Shock (%)	162 (36.8)	NA	
Respiratory failure (%)	340 (77.3)	NA	
Length of hospital stay, mean (SD)	20.99 (22.96)	6.86 (8.97)	<0.05

The COVID-19 cohort includes all patients including “unknown.” The COVID-19–negative cohort is a historical comparison cohort as we previously published in Stevens *et al.*^20^

AKI, acute kidney injury; AKIN, Acute Kidney Injury Network; CKD, chronic kidney disease; NA, data not available; NS, not significant; SCr, serum creatinine.

### Association of uNGAL With AKI

**Figure 2:**
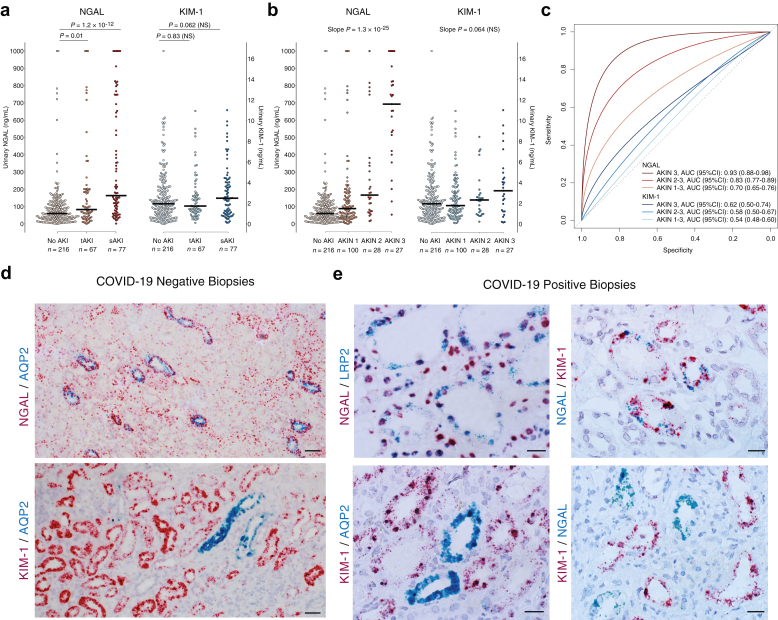
uNGAL is associated with the duration and severity of acute tubular injury in patients with COVID-19: (a) uNGAL, but not uKIM-1, was associated with sAKI (meeting AKIN criteria for ≥72 hours) in patients with COVID-19. No AKI and transient AKI (<72 hours) levels are found for comparison. (b) uNGAL, but not uKIM-1, was associated with the severity of AKI (AKIN stage); bars represent medians. Notably, mean levels of uKIM-1 were equally elevated in all 4 groups, including in patients with COVID-19 with AKIN stage 0 (no elevation of SCr). (c) ROC curves for uNGAL (shades of red) and uKIM-1 (shades of blue), by ascending AKI severity (AKIN 1–3 vs. 0, AKIN 2–3 vs. 0–1, AKIN 3 vs. 0–2). (d) In non–COVID-19 AKI biopsies, NGAL (*LCN2*) mRNA is expressed in the distal nephron including AQP2+ collecting ducts (top panel), whereas KIM-1 (*HAVCR1*) is expressed in proximal tubules and not in AQP2^+^ collecting ducts (bottom panel). Bars = 50 μM. (e) COVID-19–positive kidney biopsies with widespread acute tubular injury (80%–100% of tubules) had extensive expression of NGAL in a noncanonical distribution, in LRP2^+^ and KIM-1^+^ proximal tubules (top panels), whereas COVID-19 kidney biopsies with limited ATI (30% of tubules) exhibited limited NGAL-KIM-1 overlap (bottom right panel). KIM-1 was expressed only in AQP2^−^ proximal tubules (bottom left panel). Tissue sections were counterstained with hematoxylin. Bars = 20 μM. AKI, acute kidney injury, AKIN, Acute Kidney Injury Network; ATI, acute tubular injury; NS, not significant; ROC, receiver operating characteristics; sAKI, sustained acute kidney injury; SCr, serum creatinine; uKIM-1, urinary KIM-1; uNGAL, urinary neutrophil gelatinase-associated lipocalin.

Admission uNGAL levels were elevated among patients who subsequently met SCr-based AKI criteria (uNGAL: 267 ± 301 vs. 96 ± 139 ng/ml, *P* < 0.0001) and among patients having sAKI (lasting ≥72 hours; sAKI; uNGAL: 332 ± 324 vs. 96 ± 139 ng/ml, *P* < 0.0001) compared with those who did not have evidence of AKI (Figure 2a, log-transformed data in Supplementary Figure S1). uNGAL levels increased in a stepwise manner with increasing AKIN stage (*P* < 0.0001) (Figure 2b, log-transformed data in Supplementary Figure S1). Similarly, the area under the receiver operating characteristics curve for uNGAL progressively increased with higher AKIN stages (0.70–0.93; Figure 2c). uNGAL had 80% specificity and 75% sensitivity to diagnose AKIN stage 2 or 3 at a cutoff level of 150 ng/ml (Tables 2 and 3). In contrast, uNGAL was found to have smaller elevations in patients who experienced transient AKI (187 ± 257 vs. 96 ± 139 ng/ml, *P* < 0.05) or the AKIN 1 stage (162 ± 219 vs. 96 ± 139 ng/ml, *P* < 0.01) compared with those without elevation of SCr (Figure 2a and b). In fact, uNGAL (<150 ng/ml) had a negative predictive value of 95% (95% CI: 92%–97%) for the subsequent development of AKIN stage 2 to 3.

**Table 2:** Diagnostic properties of urinary NGAL at test cutoffs from 100 to 250 ng/ml

Outcome	Cutoff (ng/ml)	Cutoff (z-score)	Specificity	Sensitivity	PPV	NPV	+ LR	−LR
Sustained AKI	100	0.22	0.66	0.71	0.37	0.89	2.12	0.43
	150	0.50	0.80	0.57	0.44	0.87	2.93	0.53
	200	0.69	0.88	0.45	0.51	0.86	3.88	0.62
	250	0.85	0.93	0.40	0.60	0.85	5.41	0.65
AKIN 1–3	100	0.22	0.69	0.58	0.57	0.69	1.86	0.61
	150	0.50	0.85	0.47	0.70	0.69	3.16	0.62
	200	0.69	0.93	0.37	0.78	0.67	4.94	0.68
	250	0.85	0.96	0.30	0.84	0.66	7.24	0.73
AKIN 2–3	100	0.22	0.64	0.82	0.29	0.95	2.30	0.28
	150	0.50	0.80	0.75	0.39	0.95	3.67	0.32
	200	0.69	0.89	0.67	0.51	0.94	5.89	0.37
	250	0.85	0.93	0.62	0.61	0.93	8.85	0.41
AKIN 3	100	0.22	0.62	0.96	0.17	1.00	2.52	0.06
	150	0.50	0.77	0.93	0.24	0.99	3.97	0.10
	200	0.69	0.86	0.93	0.34	0.99	6.62	0.09
	250	0.85	0.91	0.89	0.43	0.99	9.53	0.12
Dialysis	100	0.22	0.61	0.83	0.11	0.98	2.12	0.27
	150	0.50	0.74	0.75	0.15	0.98	2.93	0.34
	200	0.69	0.84	0.63	0.18	0.97	3.86	0.45
	250	0.85	0.88	0.58	0.23	0.97	5.03	0.47

+LR, positive likelihood ratio; AKI, acute kidney injury; AKIN, Acute Kidney Injury Network; −LR, negative likelihood ratio; NGAL, neutrophil gelatinase-associated lipocalin; NPV, negative predictive value; PPV, positive predictive value.

**Table 3:** Diagnostic properties of urinary NGAL test at test cutoffs corresponding to specificity of 80%, 85%, and 90%

Outcome	Cutoff (ng/ml)	Cutoff (z-score)	Specificity	Sensitivity	PPV	NPV	+ LR	− LR
Sustained AKI	150	0.50	0.80	0.57	0.44	0.87	2.88	0.53
	175	0.60	0.85	0.49	0.48	0.86	3.31	0.60
	205	0.71	0.90	0.45	0.56	0.86	4.58	0.61
AKIN 1–3	130	0.40	0.80	0.51	0.65	0.69	2.55	0.61
	150	0.50	0.85	0.47	0.70	0.69	3.16	0.62
	170	0.58	0.90	0.43	0.75	0.68	4.16	0.64
AKIN 2–3	150	0.50	0.80	0.75	0.39	0.95	3.67	0.32
	172	0.59	0.85	0.71	0.45	0.94	4.65	0.34
	207	0.72	0.90	0.64	0.52	0.93	6.26	0.40
AKIN 3	163	0.55	0.80	0.93	0.27	0.99	4.60	0.09
	185	0.64	0.85	0.93	0.33	0.99	6.23	0.09
	234	0.80	0.90	0.89	0.41	0.99	8.71	0.12
Dialysis	173	0.60	0.80	0.67	0.16	0.98	3.33	0.42
	205	0.71	0.85	0.63	0.19	0.98	4.17	0.44
	328	1.03	0.90	0.50	0.23	0.97	5.05	0.55

+LR, positive likelihood ratio; AKI, acute kidney injury; AKIN, Acute Kidney Injury Network; −LR, negative likelihood ratio; NGAL, neutrophil gelatinase-associated lipocalin; NPV, negative predictive value; PPV, positive predictive value.

The association of uNGAL with the primary outcomes was independent of age, sex, race, ethnicity, baseline creatinine, and other comorbidities and was also independent of proteinuria measured in the same urine sample (Supplementary Table S1).

We performed a subgroup analysis of 198 patients with COVID-19 who did not have evidence of AKI on presentation to the ED (AKIN 0 stage). uNGAL levels were higher in patients with AKIN 0 who subsequently developed AKIN stages 1 to 3 within 7 days of admission (*n* = 51, mean 158 ± 237 ng/ml) compared with those who did not develop AKI (*n* = 147, mean 74 ± 65 ng/ml, *P* < 0.05). In this subgroup, the association of admission uNGAL level with subsequent recognition of AKI remained significant in our multivariable model (adjusted OR [95% CI]: 1.68 per SD of uNGAL [1.06–2.80], *P* < 0.05).

**Figure 3:**
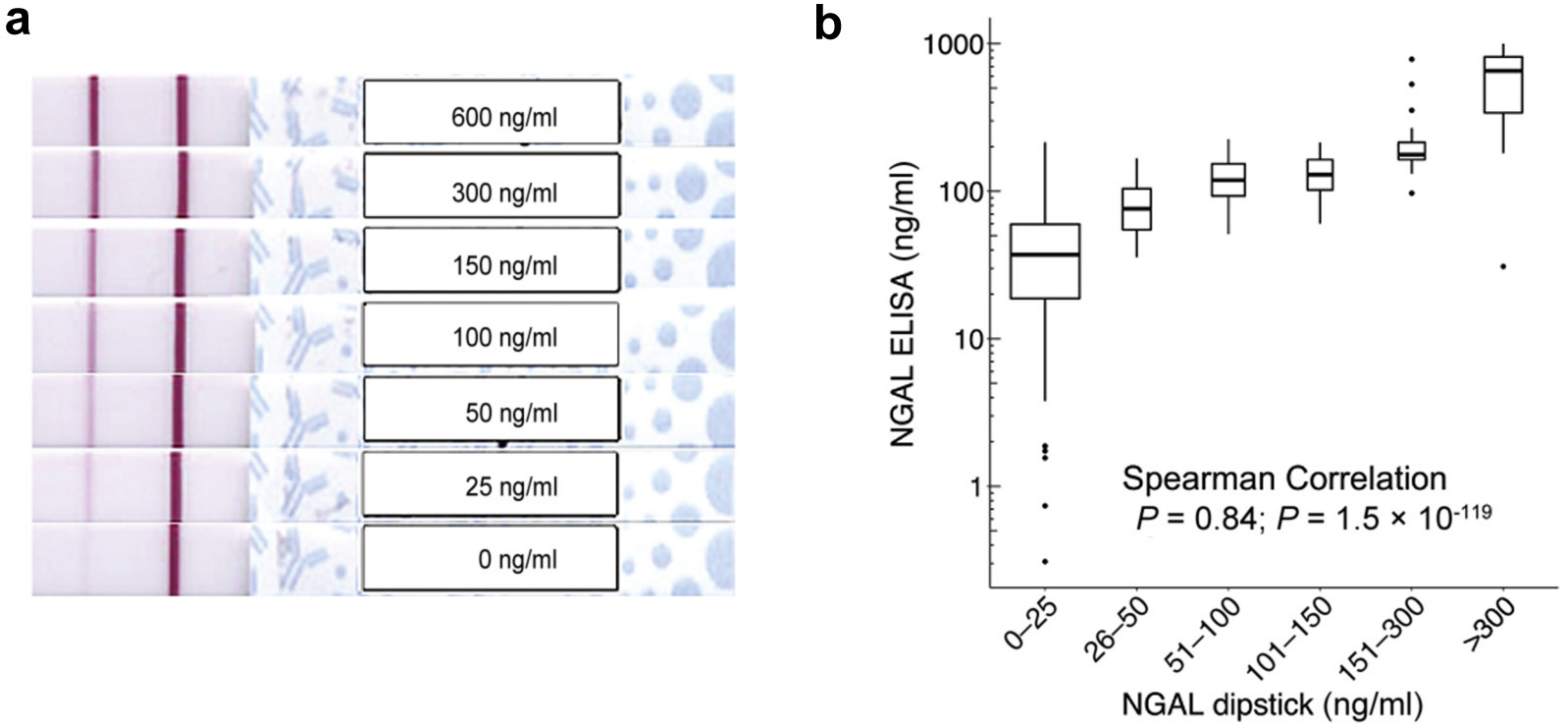
Urinary NGAL dipstick: (a) Urinary NGAL dipstick reveals dose responses to increasing concentrations of NGAL in the urine. (b) Correlation between urine dipstick and ELISA measurements. ELISA, enzyme-linked immunosorbent assay; NGAL, neutrophil gelatinase-associated lipocalin.

uNGAL level measured using a novel rapid point-of-care semiquantitative dipstick^20^ deployed to the bedside correlated with ELISA measurements (Spearman’s correlation ρ = 0.84, *P* < 0.0001) and reproduced all of the associations of uNGAL with AKI, sAKI, and AKIN stages (Figure 3a and b and Supplementary Figure S2).

### Association of uNGAL With Critical Illness

**Figure 4:**
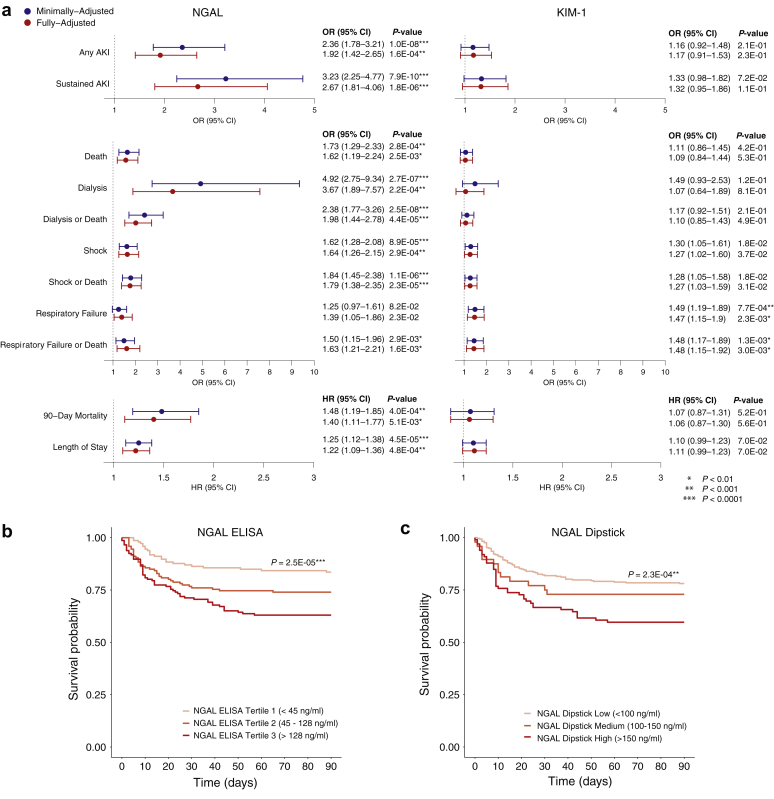
Higher urinary NGAL levels are associated with critical illness and death in patients with COVID-19: (a) Urinary NGAL levels were associated with AKI and sustained AKI (>72 hours) after adjustment for age, sex, race, and ethnicity (minimally adjusted model, blue) and baseline SCr and preexisting comorbidities (fully adjusted model, red*; n* = 371). Urinary NGAL levels were also associated with secondary outcomes of death, dialysis, shock, and respiratory failure in both minimally and fully adjusted models, *N* = 440. In contrast, uKIM-1 level was not associated with AKI or any secondary outcomes except for respiratory failure. ORs and HRs are expressed per 1 unit of SD of biomarker distribution; 95% CI. (b) Kaplan-Meier survival analysis reveals survival differences by tertile of urinary NGAL levels measured by ELISA or (c) by 3 levels of urinary NGAL dipstick test (unadjusted *P* values provided for both b and c; *N* = 440). AKI, acute kidney injury; ELISA, enzyme-linked immunosorbent assay; HR, hazard ratio; NGAL, neutrophil gelatinase-associated lipocalin; OR, odds ratio; SCr, serum creatinine; uKIM-1, urinary KIM-1.

In addition to AKI metrics, admission uNGAL level was associated with the subsequent initiation of acute dialysis (adjusted OR [95% CI]: 3.59 [1.83–7.45], *P* < 0.001), the occurrence of shock, in-hospital death (adjusted OR [95% CI]: 1.51 [1.10–2.11], *P* < 0.05) and increased overall length of hospital stay (Figure 4a), independently of demographics, comorbidities, baseline renal function, and proteinuria (Supplementary Table S1). A similar association between the level of admission uNGAL and subsequent dialysis initiation and death (minimally adjusted OR [95% CI]: 2.43 [1.47–4.29], *P* < 0.01 and fully adjusted OR [95% CI]: 2.35 [1.39–4.23], *P* < 0.01) was evident even among patients with normal SCr level on presentation (AKIN 0). In contrast, patients with lower admission uNGAL levels (<173 ng/ml) were unlikely to need dialysis (negative predictive value [95% CI]: 0.98 [0.96–0.99]).

In a time-to-event analysis, a dose-dependent association of uNGAL with 90-day mortality was observed in both ELISA and dipstick measurements. Within 30 days of admission, the highest uNGAL ELISA tertile (>128 ng/ml) and the highest uNGAL dipstick category (>150 ng/ml) both revealed lower survival probability, from 86.3% to 71.2% and from 81.8% to 66.7%, respectively, comparing the lowest and highest tertiles (Figure 4b and c).

In Supplementary Figure S3 and Supplementary Table S2, we summarized secondary outcome comparisons for patient subgroups defined by a combination of uNGAL levels (marker of tubular injury) and SCr levels (marker of functional AKI). These analyses reveal that high uNGAL levels (≥150 ng/ml) provide additional prognostic information beyond AKIN AKI criteria, consistent with our primary analysis.

In contrast to uNGAL, uKIM-1 levels were not associated with primary outcomes of AKI, sAKI, or AKIN stage in the COVID-19 cohort (Figure 2, log-transformed data in Supplementary Figure S1) nor with secondary outcomes including 90-day mortality (Figure 4a and Supplementary Table S1).

### Comparison of COVID-19 (−) and COVID-19 (+) ED Cohorts

To find whether our findings were specific to COVID-19, we evaluated a second cohort of comparable size (426 patients) admitted through the same ED (June 2017–January 2019)^20^ before the COVID-19 pandemic in New York City and analyzed using identical methods (Table 1). The COVID-19 cohort was older and enriched in Latinx patients, but the burden of chronic kidney disease was similar in both cohorts. Notably, patients with COVID-19 were 2.6 times more likely to present with AKI (35.2% vs. 13.6%, *P* < 0.0001), 3.9 times more likely to have sAKI (17.5% vs. 4.5%, *P* < 0.0001), and 1.8 times more likely to have more severe disease (AKIN 2–3, 12.5% vs. 6.8%, *P* < 0.01) compared with our historical cohort (Table 1), similar to published data.^28^

### Proximal Tubule Injury in COVID-19 Without AKI at Hospital Presentation

Urinary findings differed in the COVID-19 (−) and COVID-19 (+) cohorts even in the absence of AKI. KIM-1 was elevated in COVID-19 (+) AKIN 0 cases compared with the COVID (−) AKIN 0 historical cohort (uKIM-1: 2.57 ± 2.44 in COVID-19 [+] vs. 1.96 ± 2.51 ng/ml in COVID-19 [−], *P* < 0.01; Supplementary Figure S4). Proteinuria was also increased in COVID-19 (+) compared with COVID (−) AKIN 0 cases (*P* < 0.0001; Supplementary Figure S5). In addition, evaluation of the cellular pellets of the AKIN 0 urine samples revealed that the shedding of proximal tubule cells (marked by proximal tubule gene LRP2) was more prominent in COVID-19 (+) than in COVID-19 (−) cohort (*n* = 40; 2.61-fold increase in urinary LRP2+ cells, *P* < 0.01), whereas UMOD+ cells were present regardless of COVID-19 status (Supplementary Figure S6). In sum, AKIN 0 COVID-19 urine was enriched for KIM-1, proteinuria, and proximal tubule cells. As a result, KIM-1 was not substantially increased with progressive AKIN stages in the COVID (+) cohort, whereas uNGAL was associated with AKIN stages in both COVID (+) and COVID (−) cohorts (Supplementary Figure S4).

### NGAL RNA Expression Correlates With Histopathology

To explore the responses of different nephron segments, we evaluated the transcriptomic patterning of the biomarkers in kidney biopsies from 13 patients with COVID-19^23^ and 4 controls without COVID-19 with ATI. In both COVID-19 and non–COVID-19 biopsy findings, KIM-1 was found to be expressed in the proximal tubule whereas NGAL was prominently expressed in the limbs of Henle and collecting ducts. The distributions were confirmed by simultaneously probing for segment-specific markers, LRP2 (proximal tubule) and AQP2 (collecting duct). Surprisingly, in addition to its canonical distribution, NGAL transcripts were expressed in additional nephron segments in COVID-19 biopsy samples. At maximum extent of ATI (>50% of tubules), KIM-1 was expressed in 27% (3322 of 12,123), whereas NGAL was expressed in 65.7% (6580 of 10,111) of the tubules, including significant coexpression with KIM-1 in 85% of COVID-19 kidneys and with proximal marker LRP2 in 77% of the kidneys (Figure 2d and e). Furthermore, 62.5% of the tubules in high ATI biopsy samples and 20% of the tubules in low ATI biopsy samples (*P* < 0.05) were found to have coexpression of KIM-1 and NGAL, implying severity of ATI drives NGAL RNA patterning.

### Confirmation That NGAL Expression Correlates With Histopathology in Models of Injury

**Figure 5:**
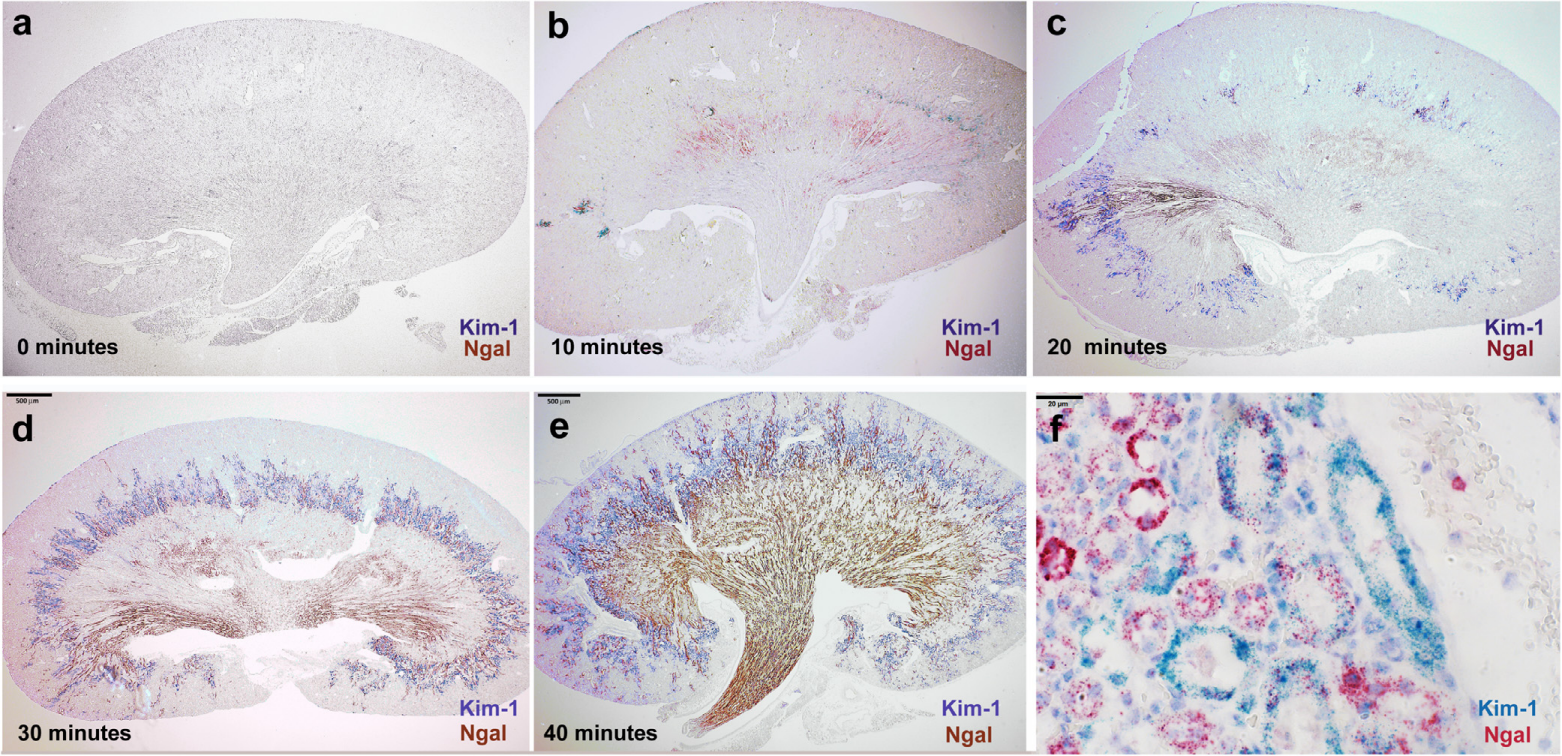
The expression level of NGAL (red-brown) and KIM-1 (blue-purple) depended on the dose of arterial ischemia in mouse: (a–e) NGAL expression was found at the corticomedullary junction after 10 minutes of ischemia, but throughout the medulla and papilla after 30 to 40 minutes of ischemia. KIM-1 expression was found in the cortex and throughout the corticomedullary junction. (e–f) Prolonged ischemia (40 minutes) broadened the expression domain of NGAL to include the proximal tubule marked by KIM-1. In contrast to NGAL, KIM-1 expression remained localized to the cortex and corticomedullary junction. Bars a–e = 500 μm; bars f = 20 μm. NGAL, neutrophil gelatinase-associated lipocalin.

To confirm that the patterning of NGAL RNA in COVID (+) biopsy samples reflected ATI, we evaluated a classical model of ischemia–reperfusion injury in the mouse. Similar to human kidneys, increasing degrees of ATI also resulted in broadening of NGAL RNA expression in mouse kidneys (Figure 5a–f). In the setting of prolonged arterial ischemia, the entirety of the corticomedullary junction, medulla, papilla, and KIM-1+ proximal tubules expressed NGAL RNA. These findings are consistent with a recent report describing NGAL expression in the proximal tubule cells by single-cell sequencing after ischemic injury^29^ and revealing the expansion of NGAL RNA in COVID (+) biopsies is a reproducible consequence of severe kidney injury.

## Discussion

We have identified a quantitative association between uNGAL and both functional kidney failure (AKI) and ATI in patients admitted to the hospital with COVID-19 infection. The level of uNGAL was associated in a stepwise fashion with clinical metrics and outcomes of AKI, such as dialysis, whereas renal biopsy findings correlated NGAL RNA with more severe forms of ATI. Consequently, admission uNGAL measurements provided prognostic data relevant to kidney injury and dysfunction in the COVID (+) patients.

As diagnostic tools, uNGAL and SCr have many different characteristics.^12^ NGAL is expressed within 2 to 3 hours of injury,^13^^,^^30^^,^^31^ whereas the elevation of SCr level is delayed by 24 to 48 hours,^32^^,^^33^ depending on mechanisms that enhance its excretion (the renal reserve) or limit its production.^34^ In addition, NGAL is detected in the urine after small wedge infarctions^13^ and unilateral kidney disease,^35^ whereas SCr is insensitive to focal or subtotal injury. In fact, we found that elevated uNGAL level was associated with AKI and clinical outcomes, even when patients with COVID-19 presented at admission without AKI (AKIN 0), confirming admission SCr level underestimated the evolution of COVID-19–associated kidney disease, revealing the differential sensitivity of the 2 analytes.

The stepwise association of an injury marker, NGAL, and a functional marker of glomerular filtration, SCr, can be explained by the progressive induction of NGAL RNA in the kidney with greater degrees of histopathologic injury (Figure 2). Increasing severity of injury broadened the classical patterning of NGAL RNA expression in the limb of Henle and collecting ducts to encompass multiple segments of the nephron from the proximal tubule to the papilla. The dose responsiveness of NGAL RNA and its patterning were reproducible beyond human biopsies to include classical models of tissue damage created by ischemia–reperfusion injury in mouse (Figure 5). Indeed, elevated uNGAL level is associated with inflammatory, ischemic, toxic, and obstructive uropathies, which injure the tubule, rather than reversible hemodynamic challenges (e.g., volume depletion, diuretics, and heart failure) that induce little, if any, response by different injury biomarkers.^12^^,^^32^^,^^36^ Hence, we reveal for the first time that the level of NGAL mirrors the severity of ATI in human kidney biopsies, including severe forms of ATI in COVID-19 kidneys. In light of this, the association of uNGAL with functional stages of AKI is likely due to its quantitative association with ATI, which in severe cases limits the clearance of SCr. The progressive increase in the area under the receiver operating characteristics curves for uNGAL, from 0.70 to 0.93 with increasing AKIN stage, highlights that severe dysfunction (AKI) is found in cases of severe injury (ATI).

The association of uNGAL with AKI and ATI provides the possibility of a sensitive diagnostic strategy that bypasses the delays and insensitivity of SCr. We reveal that accurate testing for COVID-19–associated kidney injury is possible in the ED using rapid point-of-care dipsticks.^20^^,^^37^ The NGAL dipstick correlated closely with ELISA-based measurements (ρ = 0.84, *P* < 0.0001), but the dipstick limits the risk of handling infectious body fluids. The dipstick may be particularly helpful in the setting of high patient volumes witnessed in EDs during COVID-19 surges, providing prognostic information in real time.

We also suggest that our diagnostic strategy may be further enhanced by measuring proteinuria, which is indicative of kidney injury even without elevation of SCr (AKIN 0) and is associated with a number of adverse clinical outcomes independently of NGAL. When measured together, NGAL and proteinuria may offer a comprehensive evaluation of kidney injury in COVID-19 especially in patients who have not reached criteria for AKIN staging.

Our study has a number of limitations. Similar to previous studies,^38^^,^^39^ we were limited by the use of SCr as the gold standard for AKI. Notably, many patients with COVID-19 did not have previous health records, making it difficult to establish their baseline SCr values and to detect AKI and calculate its stage. As a consequence, we were unable to define AKI in 69 patients (15.5%) with missing SCr measurements at baseline (no records) or follow-up (early death or discharge). We were also not able to collect urine samples on subsequent days because of the severity of illness, precluding comparative studies of the kinetics of urinary biomarkers, and it remains possible that subsequent measurements of uNGAL, uKIM-1, and proteinuria could have provided additional prognostic information, suggested to be important by Nugent *et al.*^40^ in understanding the impact of COVID-19

In conclusion, a large cohort of patients with COVID-19 had a dose-responsive relationship of uNGAL with ATI and AKI and severe clinical outcomes, independently of other established risk factors. These relationships were found even in patients who did not meet the SCr-based AKIN criteria in the ED. Conversely, the absence of elevated uNGAL level essentially ruled out the need for dialysis and identified patients at lower risk for death. The utility of a dipstick further underscores the value of uNGAL for rapid triage decisions. Given the recent resource challenges that the COVID-19 pandemic has created for Emergency Departments, Nephrology, and Critical Care services,^41^ uNGAL can aid in triage and disposition of patients without waiting for repetitive measurements of SCr or kidney biopsies.

## Disclosure

Columbia University owns and licensed patents involving NGAL to BioPorto and Abbott. Dr. Mohan reports receiving grant funding and personal fees from Angion Biomedica and personal fees from *KI Reports* as deputy editor. The authors have nothing else to disclose.

## Supplementary Material

Supplementary File (PDF)

**Table S1.** Urinary biomarker associations with primary and secondary outcomes (PDF).

**Table S2.** Associations of uNGAL-based stratification of AKI with clinical outcomes (PDF).

**Figure S1.** Log-transformed levels of uNGAL and uKIM-1 in patients with COVID-19 (PDF).

**Figure S2.** Comparison of urine NGAL measurements by ELISA or dipstick (PDF).

**Figure S3.** Comparison of clinical outcomes in patients stratified by uNGAL and SCr-based AKI (PDF).

**Figure S4.** Urinary NGAL and KIM-1 in COVID-19–negative and COVID-19–positive cohorts (PDF).

**Figure S5.** Comparison of proteinuria in COVID-19–negative and COVID-19–positive cohorts (PDF).

**Figure S6.** Shedding of LRP2+ proximal tubule cells into the urine in COVID-19 cases (PDF).

**STROBE Statement** (PDF)
