# Supplementary material for: Elevated Neutrophil Gelatinase-Associated Lipocalin Is Associated With the Severity of Kidney Injury and Poor Prognosis of Patients With COVID-19
Source: Kidney Int Rep. 2021 Oct 8;6(12):2979–92. doi: 10.1016/j.ekir.2021.09.005 (PMC8497954; doi:10.1016/j.ekir.2021.09.005)
Supplement: Supplementary File (PDF) [file mmc1.pdf]

Supplementary Material

Elevated NGAL is Associated with the Severity of Kidney Injury and Poor Prognosis of Patients with COVID-19

Katherine Xu, PhD[1], Ning Shang, PhD[1], Abraham Levitman, BS[1], Alexa Corker, BS[1], Satoru Kudose, MD[2], Andrew Yaeh, MD[1], Uddhav Neupane, BS[1], Jacob Stevens, MD[1], Rosemary Sampogna, MD, PhD[1], Vivette D'Agati, MD[2], Angela M. Mills, MD[4], Sumit Mohan, MD, MPH[1,3], Krzysztof Kiryluk, MD, MS[1], and Jonathan Barasch, MD, PhD[1,2]

[1]Department of Medicine, Columbia University, New York
[2]Department of Pathology, Columbia University, New York
[3]Department of Epidemiology, Columbia University, New York
[4]Department of Emergency Medicine, Columbia University, New York

Supplementary Tables:
Supplementary Table S1. Urinary biomarker associations with primary and secondary outcomes……………....Page 2
Supplementary Table S2. Associations of uNGAL-based stratification of AKI with clinical outcomes.………...Page 3

Supplementary Figures:

Supplementary Figure S1. Log-transformed levels of uNGAL and uKIM-1 in COVID-19 patients……………..Page 4
Supplementary Figure S2. Comparison of urine NGAL measurements by ELISA or Dipstick ……......…..........Page 5
Supplementary Figure S3. Comparison of clinical outcomes in patients stratified by uNGAL and SCr-based AKI....Page 6
Supplementary Figure S4. Urinary NGAL and KIM-1 in COVID-negative and COVID-positive cohorts ....Page 7
Supplementary Figure S5. Comparison of proteinuria in COVID-19-negative and COVID-19-positive cohorts..Page 8
Supplementary Figure S6. Shedding of LRP2+ proximal tubule cells into the urine in COVID-19 cases ....Page 9

	Minimally-Adjusted			Fully-Adjusted-1			Fully-Adjusted-2 (with Proteinuria)		
**Urinary NGAL**									
**Primary Outcomes**	**OR**	**95% CI**	**P-value**	**OR**	**95% CI**	**P-value**	**OR**	**95% CI**	**P-value**
AKIN 1-3	2.36	(1.78−3.21)	1.04E-08	1.92	(1.42−2.65)	1.62E-04	1.89	(1.39−2.63)	8.80E-05
AKIN 2-3	6.86	(4.24−11.7)	9.26E-14	6.89	(3.99−12.63)	4.16E-11	6.90	(3.98−12.66)	4.89E-11
AKIN 3	24	(10−71)	3.09E-10	34	(11−153)	9.61E-08	37	(11−169)	1.12E-07
Sustained AKI	3.23	(2.25−4.77)	7.85E-10	2.67	(1.81−4.06)	1.80E-06	2.55	(1.71−3.89)	8.51E-06
**Secondary Outcomes**	**OR**	**95% CI**	**P-value**	**OR**	**95% CI**	**P-value**	**OR**	**95% CI**	**P-value**
Death	1.73	(1.29−2.33)	2.83E-04	1.62	(1.19−2.24)	2.53E-03	1.51	(1.10−2.11)	1.23E-02
Dialysis	4.92	(2.75−9.34)	2.68E-07	3.67	(1.89−7.57)	2.24E-04	3.59	(1.83−7.45)	3.33E-04
Dialysis or Death	2.38	(1.77−3.26)	2.52E-08	1.98	(1.44−2.78)	4.36E-05	1.84	(1.33−2.61)	3.71E-04
Shock	1.62	(1.28−2.08)	8.90E-05	1.64	(1.26−2.15)	2.91E-04	1.60	(1.22−2.11)	7.85E-04
Shock or Death	1.84	(1.45−2.38)	1.12E-06	1.79	(1.38−2.35)	2.28E-05	1.71	(1.31−2.26)	1.35E-04
Respiratory Failure	1.25	(0.97−1.61)	8.16E-02	1.39	(1.05−1.86)	2.31E-02	1.22	(0.91−1.64)	1.91E-01
Respiratory Failure or Death	1.50	(1.15−1.96)	2.89E-03	1.63	(1.21−2.21)	1.55E-03	1.43	(1.05−1.95)	2.40E-02
**Time-to-Event**	**HR**	**95% CI**	**P-value**	**HR**	**95% CI**	**P-value**	**HR**	**95% CI**	**P-value**
90-Day Mortality	1.48	(1.19−1.85)	4.03E-04	1.40	(1.11−1.77)	5.09E-03	1.30	(1.02−1.66)	3.71E-02
Length of Hospital Stay	1.25	(1.12−1.38)	4.50E-05	1.22	(1.09−1.36)	4.80E-04	1.18	(1.06−1.32)	3.20E-03
**Urinary KIM-1**									
**Primary Outcomes**	**OR**	**95% CI**	**P-value**	**OR**	**95% CI**	**P-value**	**OR**	**95% CI**	**P-value**
AKIN 1-3	1.16	(0.92−1.48)	2.08E-01	1.17	(0.91−1.53)	2.31E-01	1.13	(0.87−1.48)	3.77E-01
AKIN 2-3	1.46	(1.04−2.11)	3.69E-02	1.35	(0.94−1.99)	1.20E-01	1.30	(0.90−1.94)	1.81E-01
AKIN 3	1.62	(1.01−2.72)	5.93E-02	1.38	(0.84−2.38)	2.30E-01	1.32	(0.79−2.32)	3.05E-01
Sustained AKI	1.33	(0.98−1.82)	7.24E-02	1.32	(0.95−1.86)	1.09E-01	1.22	(0.87−1.73)	2.61E-01
**Secondary Outcomes**	**OR**	**95% CI**	**P-value**	**OR**	**95% CI**	**P-value**	**OR**	**95% CI**	**P-value**
Death	1.11	(0.86−1.45)	4.21E-01	1.09	(0.84−1.44)	5.25E-01	1.00	(0.76−1.33)	9.89E-01
Dialysis	1.49	(0.93−2.53)	1.22E-01	1.07	(0.64−1.89)	8.10E-01	1.00	(0.59−1.79)	9.85E-01
Dialysis or Death	1.17	(0.92−1.51)	2.09E-01	1.10	(0.85−1.43)	4.86E-01	0.99	(0.76−1.31)	9.54E-01
Shock	1.30	(1.05−1.61)	1.76E-02	1.27	(1.02−1.60)	3.70E-02	1.24	(0.98−1.57)	7.67E-02
Shock or Death	1.28	(1.05−1.58)	1.89E-02	1.27	(1.03−1.59)	3.06E-02	1.21	(0.97−1.52)	1.02E-01
Respiratory Failure	1.49	(1.19−1.89)	7.66E-04	1.47	(1.15−1.90)	2.28E-03	1.30	(1.01−1.69)	4.47E-02
Respiratory Failure or Death	1.48	(1.17−1.89)	1.33E-03	1.48	(1.15−1.92)	3.00E-03	1.30	(0.99−1.70)	5.61E-02
**Time-to-Event**	**HR**	**95% CI**	**P-value**	**HR**	**95% CI**	**P-value**	**HR**	**95% CI**	**P-value**
90-Day Mortality	1.07	(0.87−1.31)	5.20E-01	1.06	(0.87−1.30)	5.57E-01	0.96	(0.78−1.19)	7.25E-01
Length of Hospital Stay	1.10	(0.99−1.23)	7.00E-02	1.11	(0.99−1.23)	7.00E-02	1.07	(0.96−1.19)	2.40E-01
**Proteinuria**									
**Primary Outcomes**	**OR**	**95% CI**	**P-value**	**OR**	**95% CI**	**P-value**	**OR**	**95% CI**	**P-value**
AKIN 1-3	1.26	(1.06−1.50)	8.21E-03	1.16	(0.96−1.39)	1.29E-01	NA	NA	NA
AKIN 2-3	1.33	(1.05−1.70)	1.87E-02	1.17	(0.91−1.51)	2.32E-01	NA	NA	NA
AKIN 3	1.46	(1.06−2.07)	2.60E-02	1	(1.00−2.00)	3.58E-01	NA	NA	NA
Sustained AKI	1.45	(1.18−1.81)	6.42E-04	1.33	(1.06−1.69)	1.61E-02	NA	NA	NA
**Secondary Outcomes**	**OR**	**95% CI**	**P-value**	**OR**	**95% CI**	**P-value**	**OR**	**95% CI**	**P-value**
Death	1.33	(1.10−1.61)	3.48E-03	1.30	(1.07−1.59)	9.74E-03	NA	NA	NA
Dialysis	1.55	(1.10−2.23)	1.47E-02	1.26	(0.86−1.86)	2.40E-01	NA	NA	NA
Dialysis or Death	1.45	(1.21−1.74)	7.60E-05	1.34	(1.10−1.63)	3.38E-03	NA	NA	NA
Shock	1.17	(1.00−1.37)	5.55E-02	1.15	(0.97−1.36)	1.04E-01	NA	NA	NA
Shock or Death	1.26	(1.08−1.47)	3.67E-03	1.22	(1.04−1.44)	1.56E-02	NA	NA	NA
Respiratory Failure	1.58	(1.30−1.95)	8.22E-06	1.65	(1.33−2.07)	8.98E-06	NA	NA	NA
Respiratory Failure or Death	1.69	(1.37−2.12)	1.88E-06	1.71	(1.36−2.17)	6.98E-06	NA	NA	NA
**Time-to-Event**	**HR**	**95% CI**	**P-value**	**HR**	**95% CI**	**P-value**	**HR**	**95% CI**	**P-value**
90-Day Mortality	1.29	(1.11−1.49)	6.16E-04	1.26	(1.09−1.47)	2.36E-03	NA	NA	NA
Length of Hospital Stay	1.18	(1.09−1.29)	1.30E-04	1.17	(1.07−1.29)	5.00E-04	NA	NA	NA

Supplementary Table S1. Urinary biomarker associations with primary and secondary outcomes: minimally-adjusted model includes age, sex, race, ethnicity as covariates; fully-adjusted-1 model includes age, sex, race, ethnicity, baseline SCr, pre-existing obesity, diabetes, hypertension, solid or hematologic transplant, cancer, cardiovascular disease, and pulmonary disease; fully-adjusted-2 (with proteinuria) model includes proteinuria from the same urine sample along with all of the covariates from the fully-adjusted model-1; log-transformed and standard normalized urinary biomarkers were tested as continuous predictors of binary outcomes using logistic regression. Cox-proportional hazards regression model was used for 90-day mortality analysis. Competing risks regression was used to derive hazards ratios for length of hospital stay with death as a competing risk. Odds ratios (OR) and hazard ratios (HR) are expressed per one unit of biomarker standard deviation

Urinary NGAL-based stratification of AKI			
**Binary Outcomes**	**OR**	**95% CI**	**P-value**
**Death**			
NGAL− AKI+ vs. NGAL− AKI−	1.80	(0.95−3.40)	6.94E-02
NGAL+ AKI− vs. NGAL− AKI−	1.73	(0.68−4.17)	2.32E-01
NGAL+ AKI+ vs. NGAL− AKI−	2.25	(1.15−4.38)	1.68E-02
**Dialysis or Death**			
NGAL− AKI+ vs. NGAL− AKI−	1.64	(0.88−3.04)	1.14E-01
NGAL+ AKI− vs. NGAL− AKI−	2.06	(0.86−4.81)	9.60E-02
NGAL+ AKI+ vs. NGAL− AKI−	3.58	(1.91−6.79)	7.66E-05
**Shock or Death**			
NGAL− AKI+ vs. NGAL− AKI−	1.43	(0.84−2.45)	1.90E-01
NGAL+ AKI− vs. NGAL− AKI−	2.29	(1.06−5.11)	3.80E-02
NGAL+ AKI+ vs. NGAL− AKI−	2.86	(1.60−5.22)	4.70E-04
**Respiratory Failure or Death**			
NGAL− AKI+ vs. NGAL− AKI−	1.95	(0.85−5.08)	1.38E-01
NGAL+ AKI− vs. NGAL− AKI−	3.30	(0.90−21.4)	1.21E-01
NGAL+ AKI+ vs. NGAL− AKI−	3.04	(1.12−10.7)	4.74E-02
**Time-to-Event Outcomes**	**HR**	**95%CI**	**P-value**
**Time to Death (90d)**			
NGAL− AKI+ vs. NGAL− AKI−	1.42	(0.86−2.37)	1.74E-01
NGAL+ AKI− vs. NGAL− AKI−	1.20	(0.59−2.45)	6.07E-01
NGAL+ AKI+ vs. NGAL− AKI−	2.13	(1.29−3.53)	3.15E-03
**Time to Discharge**			
NGAL− AKI+ vs. NGAL− AKI−	1.24	(0.90−1.69)	1.85E-01
NGAL+ AKI− vs. NGAL− AKI−	1.63	(1.03−2.58)	3.84E-02
NGAL+ AKI+ vs. NGAL− AKI−	1.86	(1.32−2.63)	4.37E-04

Supplementary Table S2. Associations of uNGAL-based stratification of AKI with clinical outcomes: NGAL+ was defined as uNGAL≥150ng/mL, NGAL− as uNGAL<150ng/mL; AKI+ was defined as meeting AKIN criteria (AKIN 1, 2, or 3), and AKI− as not meeting AKIN criteria (AKIN 0). We tested NGAL−AKI+ (N=82), NGAL+AKI− (N=32), and NGAL+AKI+ (N=73) groups as categorical predictors of binary clinical outcomes using logistic regression with NGAL−AKI− (N=183) as reference. Cox-proportional hazards regression model was used for 90-day mortality analysis and competing risks regression was used to for length of hospital stay analysis with death as a competing risk. All effect estimates (OR and HR) are expressed in reference to NGAL−AKI− group and are adjusted for age, sex, race, and ethnicity.

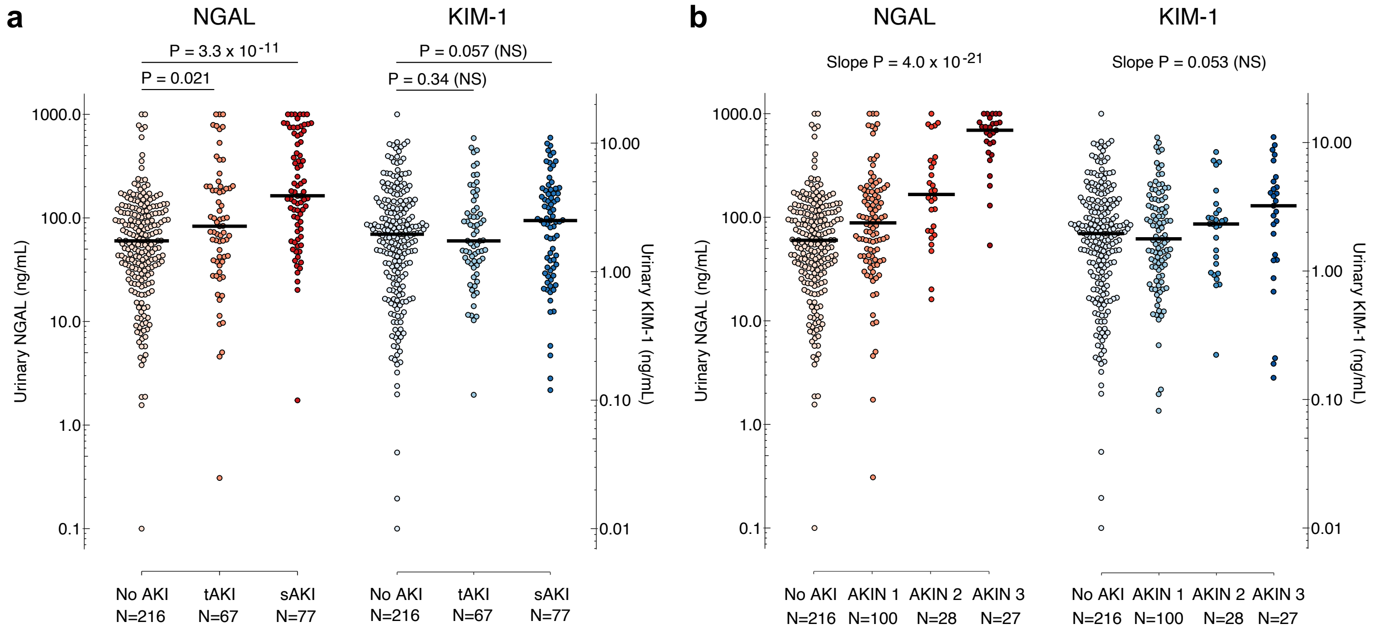

Supplementary Figure S1. Log-transformed levels of uNGAL and uKIM-1 in COVID-19 patients: uNGAL and uKIM-1 levels were log-transformed and standard-normalized for statistical testing and visualization. uNGAL is associated with the duration and severity of acute tubular injury in COVID-19 patients: (a) uNGAL, but not uKIM-1, was associated with sustained AKI (sAKI, meeting AKIN criteria for ≥72hrs) in COVID-19 patients. No AKI and transient AKI (tAKI, <72hrs) levels are shown for comparison. (b) uNGAL, but not uKIM-1, was associated with the severity of AKI (AKIN stage); bars represent medians. Notably, mean levels of uKIM-1 were equally elevated in all four groups, including in COVID-19 patients with AKIN stage 0 (no elevation of SCr).

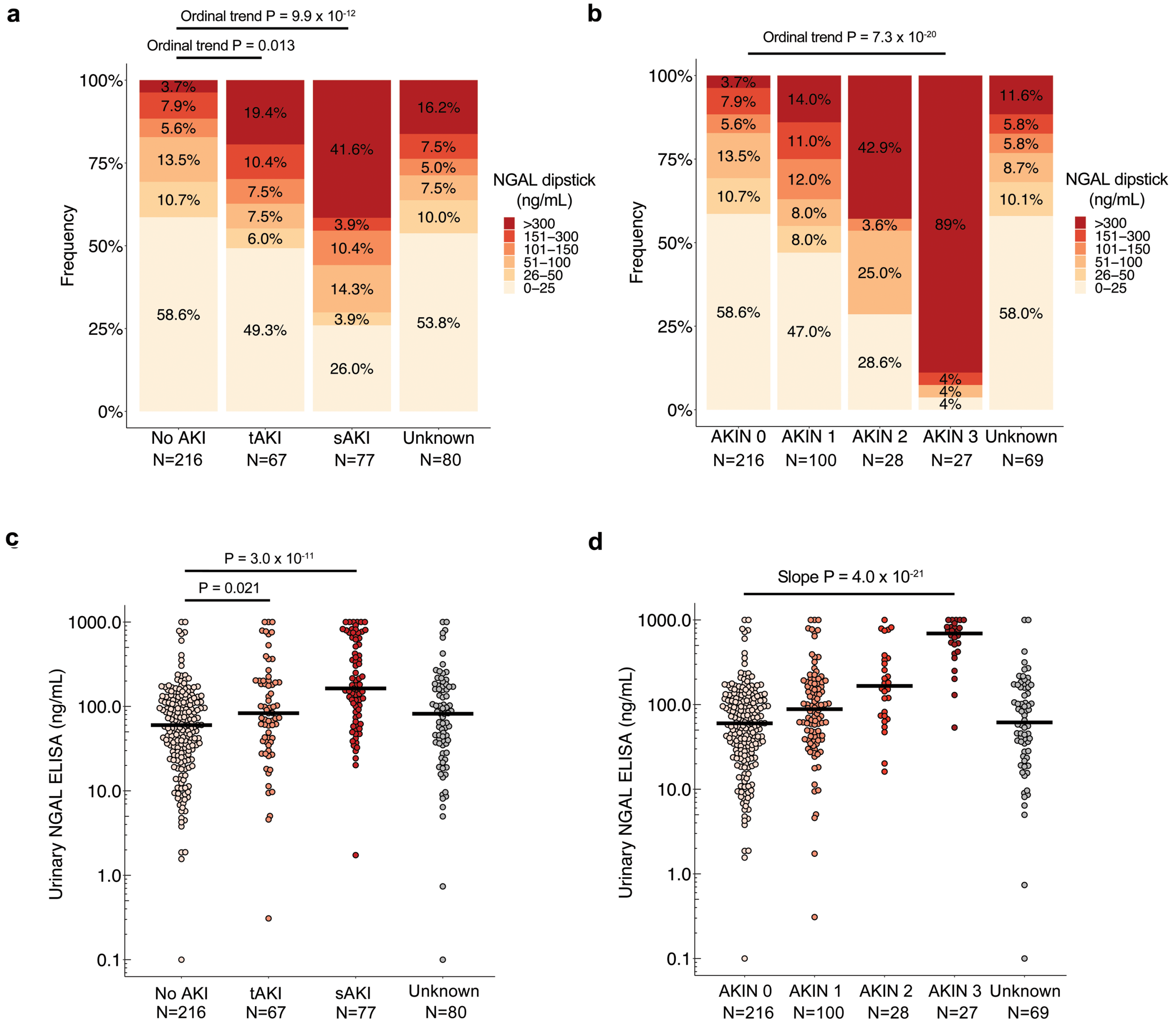

Supplementary Figure S2. Comparison of urine NGAL measurements by ELISA or Dipstick: (a-b) Urinary NGAL measured by dipstick is elevated in patients with sustained AKI and is dose-responsive to AKIN stage. **(c-d)** Urinary NGAL measured by ELISA is elevated in patients with sustained AKI and is dose-responsive to AKIN stage. Patients in the "Unknown" category (insufficient data for AKI or AKIN classification) are depicted but they are excluded from statistical analyses. Bars represent medians.

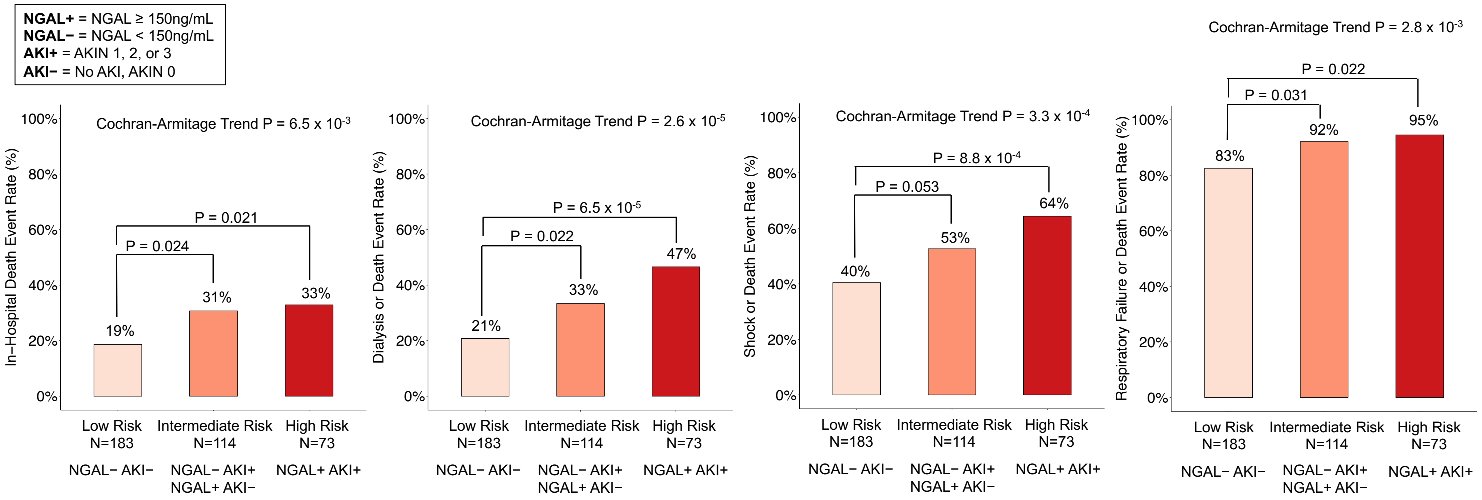

Supplementary Figure S3. Comparison of clinical outcomes in patients stratified by uNGAL and SCr-based AKI: Proportions with a clinical event (in-hospital death, dialysis or death, shock or death, and respiratory failure or death) were assessed in patients stratified by uNGAL and SCr-based AKI into the following risk groups: Low Risk = patients meeting neither high uNGAL threshold of 150ng/mL nor AKIN criteria (NGAL− AKI−, N=183), Intermediate Risk = patients meeting either high uNGAL level ≥ 150ng/mL or AKIN criteria, but not both (NGAL− AKI+ or NGAL+ AKI−, N=114), and High Risk = patients meeting both high uNGAL level ≥ 150ng/mL and AKIN criteria (NGAL+ AKI+, N=73). Cochran-Armitage test was performed to test for trend in outcome frequencies across the 3 ordered groups, and pairwise Chi-squared tests were performed to compare the Intermediate Risk and High Risk groups to the Low Risk group (NGAL− AKI− reference).

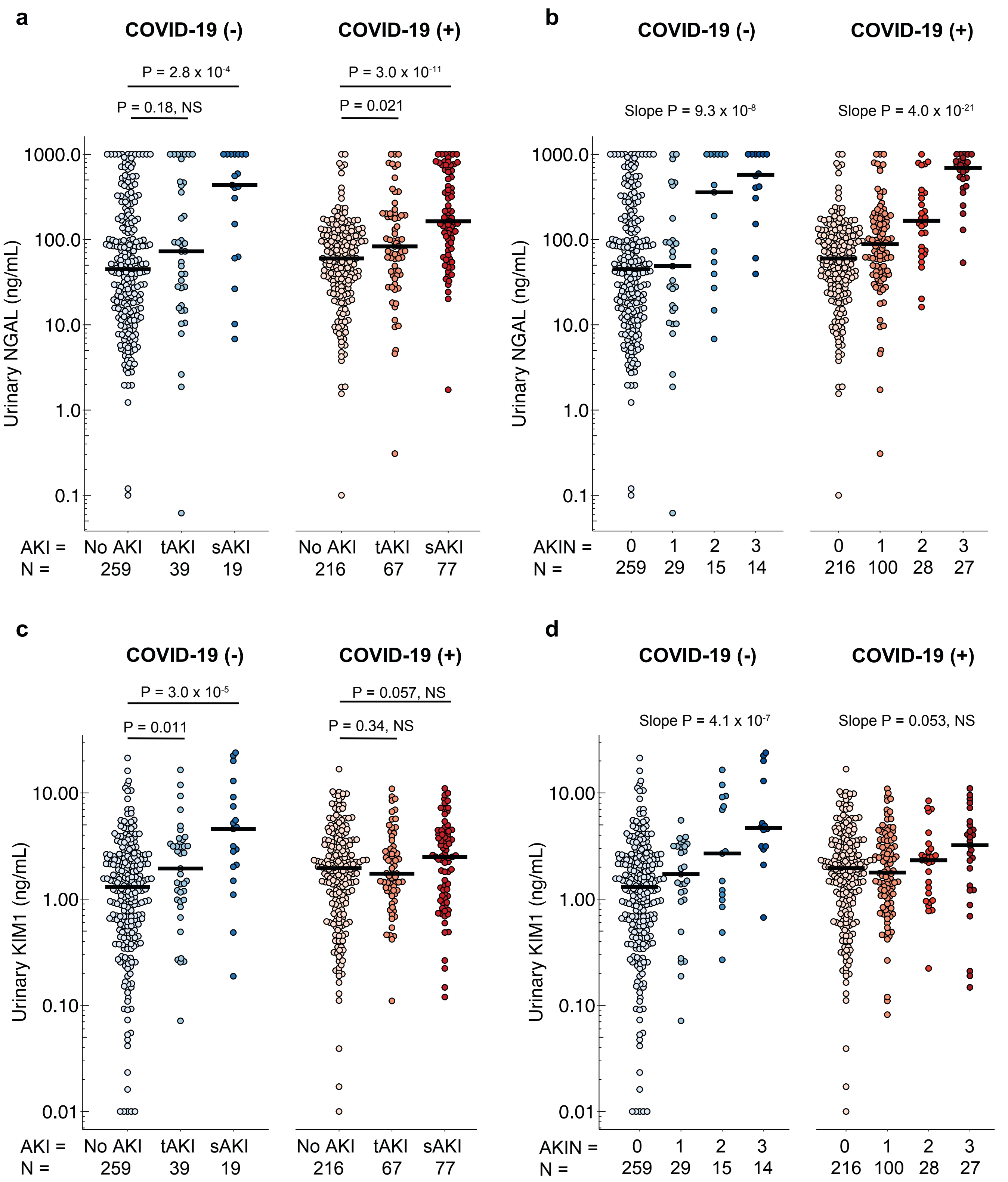

Supplementary Figure S4. Urinary NGAL and KIM-1 in COVID-negative and COVID-positive cohorts: Urinary NGAL levels by **(a)** diagnosis and duration of AKI, and by **(b)** severity of AKI. **(c)** Urinary KIM-1 levels by diagnosis and duration of AKI, and by **(d)** severity of AKI. Bars represent medians. Note that in the setting of COVID-19, uKIM-1 was elevated in all patients, including those with AKIN stage 0 (no elevation of SCr).

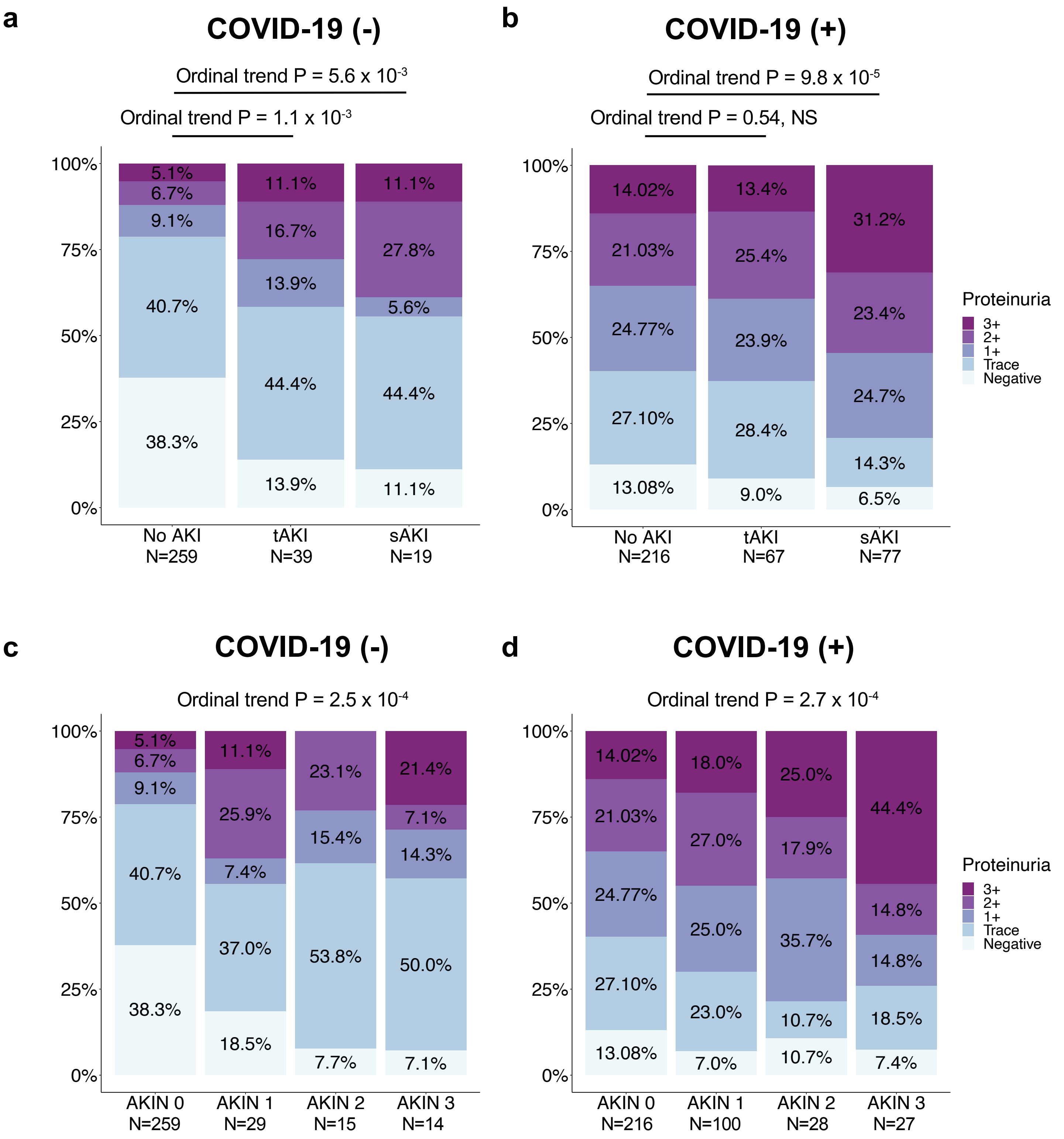

Supplmentary Figure S5. Comparison of proteinuria in COVID-19-negative and COVID-19-positive cohorts by (a-b) AKI diagnosis and duration; and by **(c-d)** increasing AKIN stage.

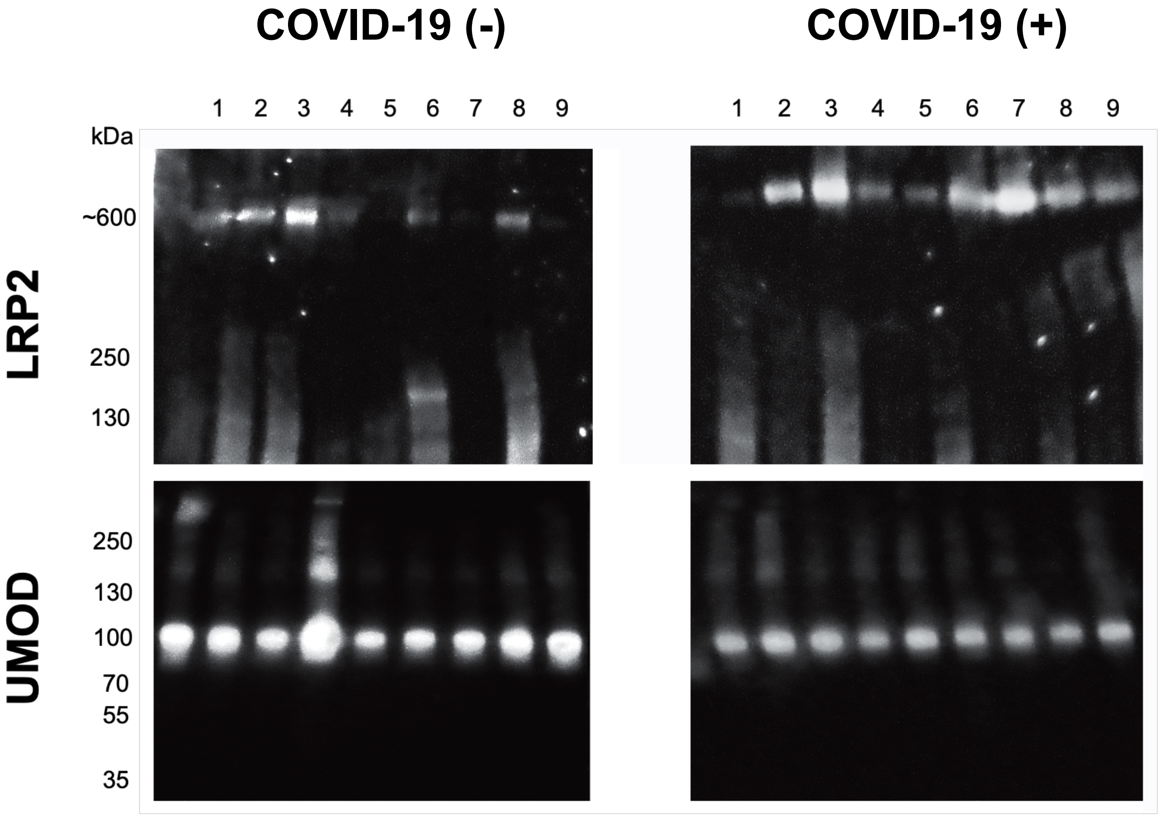

Supplementary Figure S6. Shedding of LRP2+ proximal tubule cells into the urine in COVID-19 cases: Urine cell pellets from COVID-19 positive patients without AKI (AKIN=0) demonstrated prominent LRP2+ (megalin) protein compared with urine cell pellets from non-COVID-19 patients without AKI (AKIN=0) (**top panels**). In contrast, UMOD+ (uromodulin) cells were present regardless of COVID-19 status (**bottom panels**). Molecular weights of LRP2 and UMOD are 600 kDa and 100 kDa, respectively.
